# Fractal Dimension in Healthy Hippocampi and Hippocampi With Mesial Temporal Sclerosis: An Observational Study

**DOI:** 10.7759/cureus.94110

**Published:** 2025-10-08

**Authors:** Guillermo A Gutiérrez-Aceves, Alan M Solís-Velázquez, Jesús A Rodríguez-Torres, Alejandro I Landero Sánchez, Alberto González-Aguilar, Iris E Martínez-Juárez, Alejandro Rivas-Castro, Jos A Herrera-Gonzalez

**Affiliations:** 1 Radioneurosurgery, Instituto Nacional de Neurología y Neurocirugía Manuel Velasco Suárez, Mexico City, MEX; 2 Faculty of Human Medicine "Dr. Manuel Velasco Suárez", Universidad Autónoma de Chiapas (Campus II), Tuxtla Gutiérrez, MEX; 3 Academic Unit of Human Medicine and Health Sciences, Universidad Autónoma de Zacatecas, Zacatecas, MEX; 4 Neurological Surgery, Hospital Regional “1° de Octubre” del ISSSTE (Instituto de Seguridad y Servicios Sociales de los Trabajadores del Estado), Mexico City, MEX; 5 Neuro-Oncology, Centro Oncológico Internacional, Mexico City, MEX; 6 Epilepsy Clinic, Instituto Nacional de Neurología y Neurocirugía Manuel Velasco Suárez, Mexico City, MEX; 7 Center for Educational Innovation in Medicine and Clinical Simulation, Facultad Mexicana de Medicina, Universidad La Salle, Mexico City, MEX

**Keywords:** epilepsy, fractal dimension, hippocampus, mesial temporal sclerosis, temporal epilepsy

## Abstract

A fractal is a geometric shape that has the same structure across a wide range of scales. The existence of fractal dimension has been demonstrated in various natural structures and translated into a mathematical method through which anatomical structures can be measured with high precision. The hippocampus, located in the mesial area of the temporal lobe, is affected in diseases such as epilepsy, tumors, and viral infections. Mesial temporal sclerosis (MTS) is a condition that significantly degenerates hippocampal neurons and is a major cause of drug-resistant epilepsy.

This study aimed to compare the fractal dimension between healthy hippocampi and hippocampi with MTS, and to determine whether there is a difference between them. We analyzed preoperative magnetic resonance imaging (MRI) scans from 12 patients with MTS, calculating the fractal dimension of both the diseased and the contralateral healthy hippocampus using the box-counting method in ImageJ. Healthy hippocampi had a mean fractal dimension of 1.305, while those with MTS had 1.243, representing a 4.75% reduction (p = 0.008). These results suggest that fractal dimension could serve as a quantitative biomarker for hippocampal degeneration in MTS, potentially aiding in earlier diagnosis, guiding treatment strategies, and contributing to research on structural brain changes in epilepsy.

## Introduction

“The universe is full of fractals - indeed, it might even be one itself.” A fractal is a mathematical concept defined as a geometric shape that has a detailed structure across a wide range of scales. The natural world is full of fractals; one need only think of the slopes of mountainous areas and the proliferating fronds of ferns. Fractal dimension is observed across a wide range of scales in the universe, from physical and molecular systems to anatomical structures [1].

Benoit Mandelbrot proposed fractal geometry, which was initially called the theory of “pathological” curves. It allows us to quantify terms such as irregular, rough, and complex, which are commonly found in nature and the human body. It idealizes the complexity of physical structures to understand their detailed structure at all scales and magnifications. Fractals have four properties: irregular shape, self-similarity of their structures, fractional (fractal) dimension, and scale-dependence - meaning that the measured properties depend on the scale at which they are measured. Fractal dimension has long been used to evaluate natural structures, from molecular structures to anatomical structures such as the brain [2]. Self-similarity means that similar features are found at smaller scales as the structure is magnified, so its parts, at all dimensions, resemble the whole. Unlike Euclidean geometry, fractal dimensions are expressed in fractional numbers. It reflects the properties of fractal scale - how the structure changes as it is magnified [3].

The existence of fractal dimensions in the hippocampus has been demonstrated through magnetic resonance imaging (MRI) studies [4]. In addition, recent high-resolution histological studies of the human hippocampus, using 3D phase-contrast computed tomography, have revealed complex multiscale cytoarchitectural patterns consistent with the principles of fractal organization [5]. Complementary studies in animal models have applied fractal analysis to CA1 pyramidal neurons, demonstrating that fractal dimension quantifies morphological irregularities at the cellular level [6]. These findings indicate that fractal properties are not limited to macroscopic imaging but extend into the microscopic histological domain. Fractal geometry allows us to quantitatively describe anatomical regions such as the hippocampus. The hippocampus represents a critical structure in the brain, readily visualized with high-resolution MRI and suitable for quantitative evaluation using fractal analysis. The most common neuropathological pattern found in drug-resistant epilepsy is hippocampal sclerosis (HS), which is a key feature in the mesial temporal lobe epilepsy (TLE) syndrome. Mesial temporal sclerosis (MTS) is the most common cause of medically refractory TLE and is, therefore, a frequent indication for surgical treatment. The histopathological hallmark of HS is the loss of segmental pyramidal cells with a severe pattern of astrogliosis, leading to hippocampal atrophy, and neurodevelopmental aspects may also be involved [7,8].

Surgical treatment improves patients' quality of life by reducing seizure frequency, either totally or partially. Although MTS affects other regions of the brain both metabolically and anatomically, imaging findings in the hippocampus are characteristic of diagnosis and serve as a reflection of disease progression, playing a central role in its pathogenic mechanisms [9-12].

Some specific alterations of the fractal geometry of the hippocampus could help identify pathological processes in the anatomical region, such as MTS, at an early stage - as has been demonstrated in other diseases [13,14]. The clinical gap is that hippocampal volumetry on MRI, while useful, fails to detect topological and complexity alterations that may precede or extend beyond atrophy. In TLE/MTS, hippocampal radiomics based on T2-FLAIR (fluid-attenuated inversion recovery) texture and heterogeneity discriminates patients from healthy controls, indicating that complexity-oriented metrics capture subtle pathological signals where volumetry is less informative [15]. Moreover, geometry-based pointwise analysis reveals morphometric alterations not captured by volumetry [16]. Finally, the hippocampus’ multiscale cytoarchitectural organization, demonstrated by 3D virtual histology, provides a biological rationale for applying fractal dimension to this structure [5]. Collectively, fractal dimension is potentially superior to volumetry for evaluating MTS because it measures topology-dependent complexity, is sensitive to microstructural change, and adds complementary information beyond volume [5,15,16].

The objective of this study was to compare the fractal dimension of healthy and MTS-affected hippocampi on MRI, to evaluate whether fractal analysis may serve as a quantitative biomarker of hippocampal degeneration.

## Materials and methods

We analyzed preoperative MRI scans from patients diagnosed with TLE epilepsy surgery. Surgical resection confirmed the presence of MTS in the affected hippocampus. Patients with other structural lesions, such as tumors, vascular malformations, or traumatic injuries, as well as those with poor-quality MRI images or incomplete clinical data, were excluded. The contralateral hippocampus was considered healthy only when no radiological signs of sclerosis were present, including the absence of atrophy, T2 or FLAIR hyperintensity, and preserved internal architecture. Patients with evidence of bilateral hippocampal involvement were excluded. Fractal dimension analysis was then performed on both the diseased hippocampus and the contralateral healthy hippocampus within the same patients, using the unaffected side as an internal control. The final sample comprised 12 patients, reflecting all cases that fulfilled the selection criteria within the study period. Institutional ethics approval was granted, and all datasets were anonymized prior to processing. Segmentation of hippocampal regions was conducted by a trained observer, blinded to clinical information, ensuring unbiased delineation of the structures.

A total of 12 MRI scans were evaluated. These were 3D SPGR (spoiled gradient recalled echo) image series acquired on a GE Signa 3.0 T scanner (General Electric, Boston, MA, USA) with a matrix size of 512 × 512 × 120, a field of view of 240 mm, voxel dimensions of 0.47 mm × 0.47 mm × 1.2 mm, a repetition time of 13 ms, and an echo time of 5.6 ms. From these scans, 24 hippocampi were obtained - 12 right and 12 left - which were categorized into two groups: 12 diseased and 12 healthy. The MRI scans, imported in DICOM (Digital Imaging and Communications in Medicine) format, were processed using OsiriX® software (Pixmeo, Geneva, Switzerland), where the hippocampal regions were delineated according to the Radiation Therapy Oncology Group (RTOG) guidelines (RTOG 0933) [11].

Following this, image processing was conducted using ImageJ software to prepare the images for quantitative comparison [12]. The outlined DICOM series was imported into ImageJ as a sequence. We then applied the "Subtract Background" function to the entire image sequence. Subsequently, the "Color Threshold" was adjusted with the following settings: Hue set to 1, threshold color set to black and white, and the dark background option was disabled.

**Figure 1:**
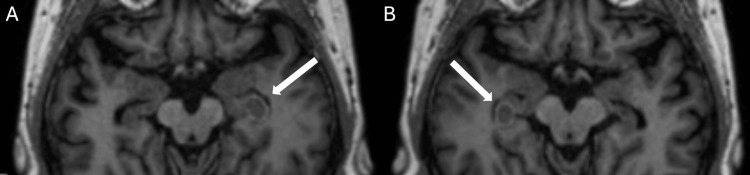
Axial Cranial MRI With Contouring of the Hippocampus in Different Slices A) Left hippocampus and B) Right hippocampus MRI, magnetic resonance imaging

Finally, the contour of the hippocampus was extracted from the original MRI series and converted into a binary object using ImageJ’s "Make Binary" option (Figure 1). This processing step was essential, as it isolated a measurable element from the system, creating a mathematical object - a quantifiable curve that represents the hippocampus for experimental analysis.

Fractal analysis was conducted using the box-counting method in ImageJ with the BoneJ plugin. This approach applies the classical mathematical model of fractal analysis, expressed as $L(x) \sim kx^{1 - D}$, adapted specifically for digital image processing. In this context, “D” corresponds to the fractal dimension of the outlined hippocampal structure. To assess the statistical significance of differences in fractal dimension between the diseased and healthy groups, a two-tailed Student’s t-test was subsequently performed.

## Results

The analysis revealed significant differences in the fractal dimension between the healthy and affected hippocampi within the same patients diagnosed with MTS. As shown in Table 1, the healthy hippocampi exhibited a mean fractal dimension of 1.305, with a standard error of 0.0167, whereas hippocampi with MTS had a lower mean of 1.243, with a standard error of 0.0179. This represents a 4.75% reduction in fractal complexity in the diseased group.

**Table 1:** Patient Characteristics and Hippocampal Fractal Dimensions SD: Standard Deviation; SEM: Standard Error of the Mean; MTS: Mesial Temporal Sclerosis

Characteristic	Value
Patients, n	12
Sex, n (female/male)	5/7
Age, years (mean ± SD)	57.1 ± 7.6
Age range, years	50-71
MTS laterality, n (left/right)	6/6
Fractal dimension - healthy hippocampus (mean ± SD)	1.305 ± 0.058
Fractal dimension - healthy hippocampus (SEM)	0.0167
Fractal dimension - healthy hippocampus (range)	1.232-1.399
Fractal dimension - MTS hippocampus (mean ± SD)	1.243 ± 0.062
Fractal dimension - MTS hippocampus (SEM)	0.0179
Fractal dimension - MTS hippocampus (range)	1.173-1.320

The statistical significance of this difference was confirmed by a two-tailed Student’s t-test (Table 2), which reported a mean difference of 0.061 (p = 0.008), indicating a highly significant result. The 95% confidence interval ranged between 0.018 and 0.106, supporting the conclusion that the decrease in fractal dimension is unlikely to be due to chance.

**Table 2:** Results of t-test

Parameter	Value
h1 = Healthy hippocampus (Sample 1)	
h2 = Diseased hippocampus (Sample 2)	
Difference in mean fractal dimension between h1 and h2	0.061946
Test result (Healthy vs. Diseased comparison)	
t-value	2.9193
p-value	0.008254

**Figure 2:**
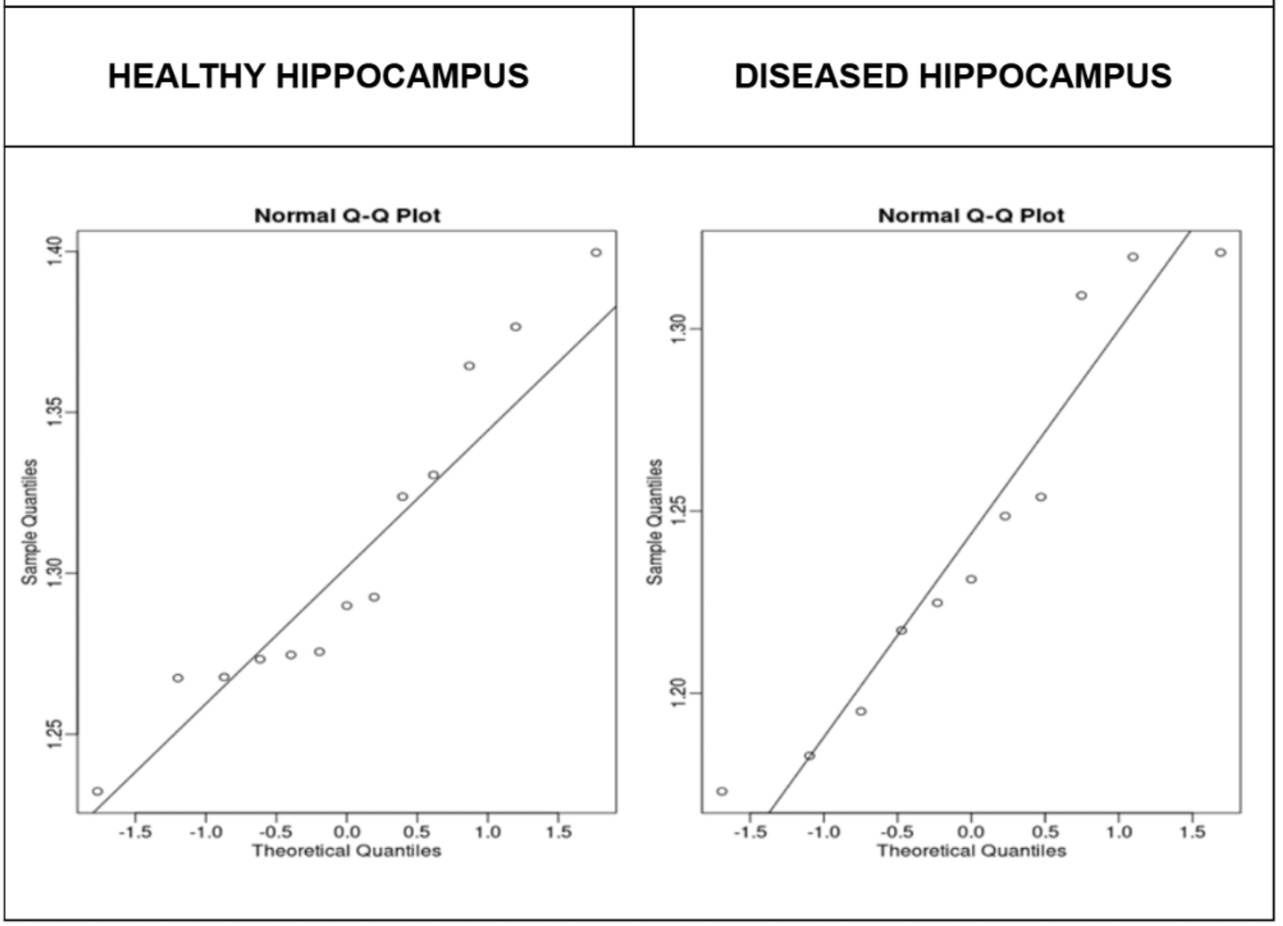
Sample Q-Q Plots (Shown With R) In the Q-Q plots, the healthy hippocampus panel shows points closely aligned with the diagonal, consistent with approximate normality and limited tail dispersion. The diseased hippocampus panel departs more clearly from the line, particularly in the tails, indicating asymmetry and higher variability. Taken together, normality is acceptable in the healthy group, whereas the diseased group shows heterogeneous fractal-dimension values.

**Figure 3:**
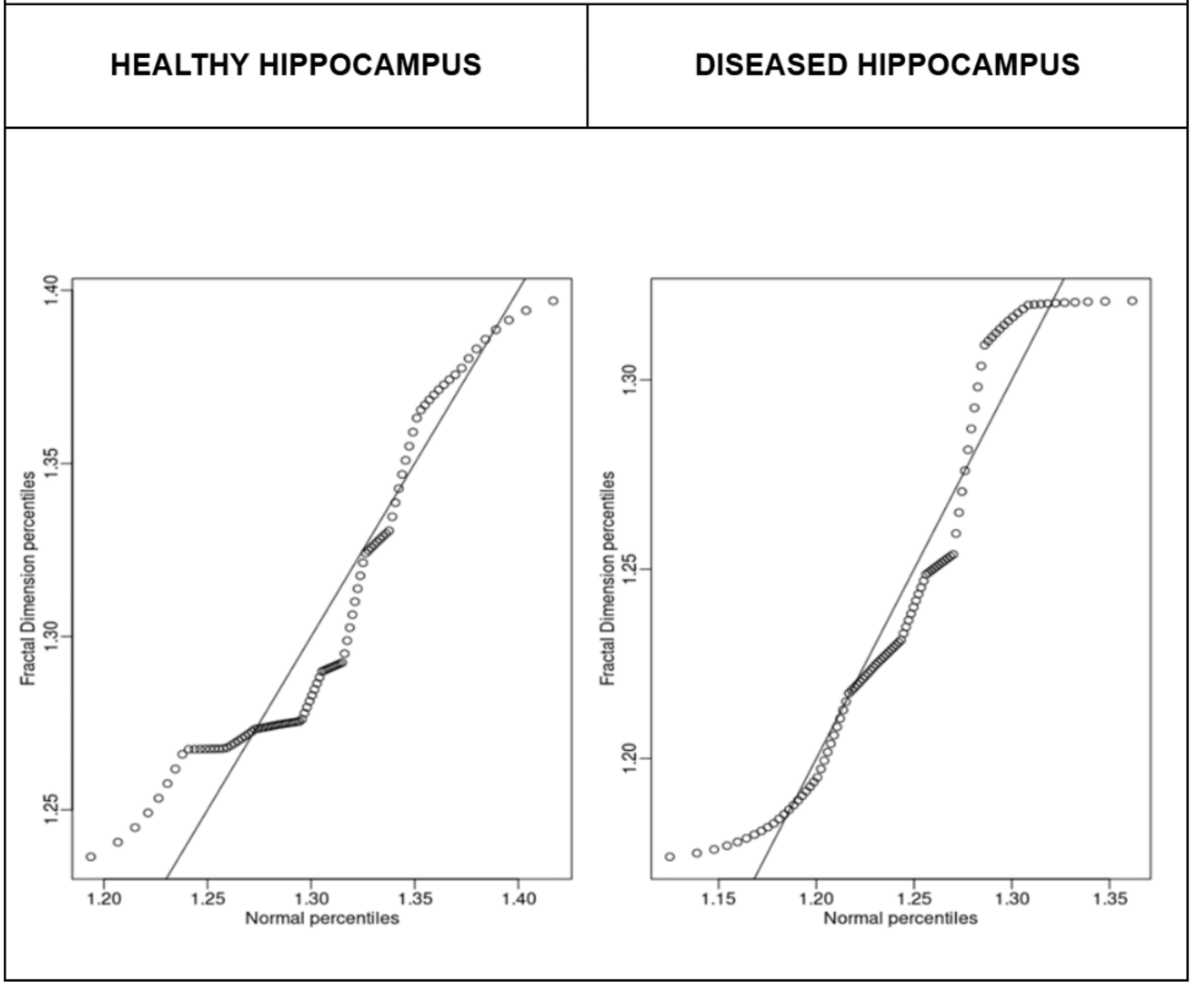
Sample Percentile Plots (Shown With R) The percentile plots reinforce this pattern: the healthy group follows an almost linear relationship with the normal percentiles, while the diseased group shows curvature and stretches, with wider point spacing especially at the lower end of the distribution. This indicates greater structural heterogeneity, precisely at low fractal-dimension values, consistent with uneven tissue involvement.

**Figure 4:**
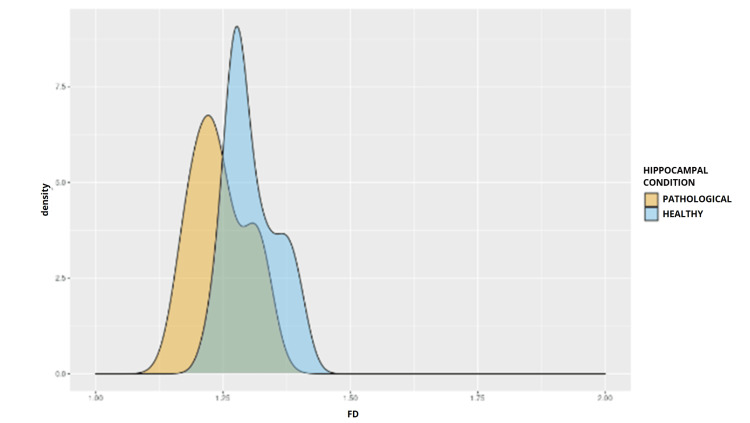
Distribution of Fractal Dimension in Healthy and Diseased Hippocampus Groups The density curve for the diseased group is shifted leftward relative to the healthy group, and concentrates more probability mass at lower values. This shift indicates an overall reduction in fractal dimension in disease, and, despite partial overlap between curves, supports the quantitative difference observed between groups.

Data normality in the healthy group was verified by Q-Q plot graphs (Figure 2) and percentile plot graphs (Figure 3). In contrast, greater dispersion was observed in the MTS group, particularly at lower values, such as the 1.15 percentile, suggesting heterogeneity in the structural involvement of the hippocampus. Furthermore, the density plot demonstrated a leftward shift of the curve in the MTS group (Figure 4), consistent with the observed reduction in fractal dimension. Together, these findings support the hypothesis that MTS reduces the fractal complexity of the hippocampus, likely due to pathological changes such as atrophy or gliosis.

## Discussion

MTS is a neuropathological alteration of structural origin, characterized by neuronal loss and gliosis in specific regions of the hippocampus - primarily in CA1, CA3, and the hilus of the dentate gyrus - especially in adults [17]. This structural cause predominates in multiple series and systematic reviews, including Latin American populations, where MTS represents the largest proportion of drug-resistant epilepsy cases, followed by other etiologies such as genetic, metabolic, inflammatory, and infectious alterations [17,18]. It is usually diagnosed by MRI, where unilateral hippocampal atrophy, T2 or FLAIR hyperintensity, and loss of normal structural differentiation are observed [5,6]. Ultimately, although the definitive diagnosis is histopathological, the combination of clinical findings (partial seizures with aura, memory disturbances, and electroencephalography (EEG) with epileptiform activity in the anteromedial temporal region) and imaging studies allows for a reliable and non-invasive confirmation of MTS [19].

In recent years, it has been described that the hippocampus, due to its morphological complexity, exhibits fractal characteristics, as we have previously reported [4]. In the present study, we propose going one step further: to objectively measure that fractal complexity using a reproducible mathematical analysis to quantify differences between healthy hippocampi and those affected by MTS. Unlike volumetric techniques, which may be influenced by imaging artifacts, operator-dependent segmentation, or the limitations of atlas-based automated algorithms in the presence of pathological changes, fractal analysis focuses on the intrinsic geometric complexity of hippocampal contours. This reduces reliance on manual delineation and may offer greater robustness in scenarios where volumetry is confounded by structural irregularities [4,13,14,20]. In clinical practice, hippocampal volumetry can be limited when atrophy is subtle or absent, so fine-scale morphometric alterations are not captured by volume [16]. In these contexts, advanced biomarkers have shown higher sensitivity: hippocampal radiomics based on T2 FLAIR differentiates patients with mesial TLE with HS from healthy controls [15]. Likewise, hippocampal T2 relaxometry can lateralize TLE and detect pathology when clinical MRI does not show overt atrophy [21]. Moreover, geometry-based pointwise morphometry identifies alterations not captured by volumetry, providing local spatial resolution [16]. Against this backdrop, fractal dimension offers a complementary index of geometric complexity that can be useful when volume is normal or ambiguous, and can be integrated with these biomarkers for a more comprehensive assessment [2].

Furthermore, the clinical utility of fractal dimension could be enhanced through integration into predictive models of surgical outcomes. Kuroda et al. found that interictal electrophysiological biomarkers significantly improve epilepsy surgery outcome prediction [22]. Sun et al. developed a genetic neural network model incorporating a hybrid intracranial EEG (iEEG) marker, boosting predictive accuracy from 87% to 94.3% [23]. Additionally, Cao et al. reviewed dynamical network analyses using non-invasive EEG and magnetoencephalography (MEG) to characterize the epileptogenic zone and guide surgical decision-making [24]. In this framework, hippocampal fractal dimension, by objectively quantifying structural complexity, could serve as a valuable complementary biomarker for surgical candidate selection and postoperative response prediction.

The results obtained from this study show a statistically significant difference in the fractal dimension of the hippocampus between healthy individuals and those with MTS, with a 4.75% reduction observed in the diseased group. This finding suggests that the structural complexity of the hippocampus, quantified through fractal analysis, is altered in the presence of MTS. Statistical results confirmed this difference. The average fractal dimension of healthy hippocampi was 1.305, a value very similar to that reported in previous studies [4], which found a mean of 1.326. In contrast, diseased hippocampi showed a lower fractal dimension in most cases, consistent with previous research where a decrease in fractal dimension has been observed in structures affected by pathological processes [13,14]. This reduction could be related to atrophy, neuronal loss, and gliosis characteristic of MTS, and reinforces the value of fractal analysis as a quantitative tool to evaluate structural alterations in the hippocampus from a mathematical and reproducible perspective [7,8].

Our cohort ranged from 50 to 71 years, with a mean of 57.1 and a standard deviation of 7.6. In this life stage, age-related declines in hippocampal volume have been described, with subfield-specific trajectories [25]. In healthy populations, cortical complexity estimated by fractal dimension decreases with advancing age [2]. Large studies and meta-analyses have documented progressive hippocampal volume loss across adulthood, with subfield-specific patterns [26]. These patterns indicate that age may confound the interpretation of the observed reduction of 4.75% in fractal dimension in MTS. We recommend adjusting for age and for other covariates, such as sex, intracranial volume, and scanner type, in regression models or linear analysis of covariance, and performing age-stratified sensitivity analyses to confirm robustness. Multimodal evidence shows that normal aging affects morphometry, connectivity, and microstructure across hippocampal subfields, reinforcing the need to include age as a covariate when evaluating complexity-based biomarkers derived from fractal dimension [27].

Despite the significant findings, our study presents some limitations that should be considered when interpreting the results. Firstly, the small sample size (24 hippocampi in total) limits the generalizability of the results to larger populations. This limitation could influence statistical variability and reduce the power to detect more subtle differences in fractal dimension between clinical subgroups, such as different degrees of MTS or specific lateralization of the involvement. Likewise, the hippocampus segmentation process, although performed following RTOG guidelines and using standardized software, could be subject to interobserver variability.

Another possible limitation is that the fractal dimension, although mathematically robust, may be influenced by the resolution and quality of the images, which could generate systematic biases if MRI acquisition is not fully standardized. Furthermore, potential associated clinical factors, such as epilepsy duration, seizure burden, previous treatment, etc., were not explored, which could have correlations with the observed structural complexity. Our results support the potential of fractal dimension as a quantitative biomarker of structural damage in the hippocampus in the context of MTS. However, future studies with larger samples and greater clinical diversity are necessary to confirm the reproducibility of these findings.

In particular, multicenter research could explore whether fractal dimension varies according to factors such as age of seizure onset, lateralization of the epileptic focus, or response to surgical treatment. In addition, it would be relevant to evaluate the correlation between fractal dimension and clinical or neuropsychological measures, such as memory test performance, seizure frequency, or quality of life. This methodology could also be applied to other pathologies affecting the hippocampus, such as Alzheimer's disease or limbic encephalitis. It would also be valuable to develop automatic fractal analysis tools integrated into imaging workstations, which would allow greater standardization and clinical applicability of the method. This could facilitate inclusion in diagnostic algorithms.

## Conclusions

We observed objective differences in fractal dimension between healthy and diseased hippocampi. Quantitatively, the fractal dimension was lower by 4.75% in hippocampi with MTS compared with healthy controls. Although the sample size was limited, these findings offer strong preliminary evidence supporting the potential diagnostic value of fractal dimension, particularly in conditions where anatomical changes are subtle or difficult to detect using conventional methods. Fractal dimension analysis provides an objective, quantitative tool for assessing hippocampal structural complexity and can complement volumetric measurements in clinical evaluation and treatment planning. Beyond its diagnostic role, fractal analysis may also facilitate earlier detection of hippocampal pathology and provide complementary information for treatment planning in patients with MTS. Future studies, involving larger populations, greater clinical diversity, and functional correlations, could help establish this metric as a complementary biomarker in the evaluation of hippocampal pathologies, foremost of which is MTS.
